# Implantation of a capsular tension ring during cataract surgery attenuates predicted remodeling of the post-surgical lens capsule along the visual axis

**DOI:** 10.3389/fbioe.2023.1300830

**Published:** 2024-01-19

**Authors:** Kurt A. Ameku, Caleb C. Berggren, Ryan M. Pedrigi

**Affiliations:** Department of Mechanical and Materials Engineering, University of Nebraska-Lincoln, Lincoln, NE, United States

**Keywords:** finite element model, growth and remodeling, biomechanics, mechanobiology, intraocular lens, posterior capsule opacification

## Abstract

**Introduction:** Cataract surgery permanently alters the mechanical environment of the lens capsule by placing a hole in the anterior portion and implanting an intraocular lens (IOL) that has a very different geometry from the native lens. We hypothesized that implant configuration and mechanical interactions with the post-surgical lens capsule play a key role in determining long-term fibrotic remodeling.

**Methods:** We developed the first finite element-growth and remodeling (FE-G&R) model of the post-surgical lens capsule to evaluate how implantation of an IOL with and without a capsular tension ring (CTR) impacted evolving lens capsule mechanics and associated fibrosis over time after cataract surgery.

**Results:** Our models predicted that implantation of a CTR with the IOL into the post-surgical lens capsule reduced the mechanical perturbation, thickening, and stiffening along the visual axis in both the remnant anterior and posterior portions compared to implantation of the IOL alone.

**Discussion:** These findings align with patient studies and suggest that implantation of a CTR with the IOL during routine cataract surgery would attenuate the incidence of visually-debilitating capsule fibrosis. Our work demonstrates that use of such modeling techniques has substantial potential to aid in the design of better surgical strategies and implants.

## 1 Introduction

Cataracts is the leading cause of blindness worldwide (Wormstone et al., 2021) and the corrective procedure is the most commonly performed eye operation in the world with approximately 20 M procedures annually (Day et al., 2016). Cataract surgery involves placing a hole in the anterior lens capsule, known as a continuous circular capsulorhexis (CCC), breaking up and removing the opacified lens fibers, and implanting an intraocular lens (IOL). An interesting feature of this procedure is that the CCC is permanent. We have previously hypothesized that this permanent mechanical perturbation drives the long-term errant response of the lens epithelial cells after surgery (Pedrigi et al., 2007; Pedrigi et al., 2009a; Pedrigi and Humphrey, 2011). In particular, lens epithelial cells differentiate into a wound-healing myofibroblast phenotype that causes them to become proliferative, synthetic, contractile, and migratory (Lovicu et al., 2016). These fibrotic behaviors are particularly focused around the CCC edge (which can cause anterior capsule opacification or ACO), the IOL haptics at the equator, and, eventually, the posterior capsule due to cell migration. Here, cell deposition of matrix proteins and contraction of the capsule can lead to the formation of posterior capsule opacification (PCO) which causes the patient to experience particularly severe visual disturbances (Wormstone et al., 2021). An approach that has been successful at mitigating PCO, though not eliminating it, is the use of IOLs with a square-edged optic that physically inhibits epithelial cell migration to the posterior capsule (Wormstone et al., 2021). This demonstrates the importance of the mechanical interaction between the capsule and implant in determining PCO.

Another device that is sometimes implanted during cataract surgery along with the IOL is a capsular tension ring (CTR). This is most often done when patients have experienced trauma or have an underlying condition, such as pseudoexfoliation syndrome, that causes weakness of the zonular fibers that connect the lens to the ciliary muscle and hold it in place within the eye (this connection is also central to the process of accommodation of the native lens). Several studies have concluded that CTRs improve IOL stability within the remnant capsular bag and reduce the incidence of IOL decentration (Li et al., 2016; Miyoshi et al., 2020), capsule contraction (Chen et al., 2021; Yang et al., 2021), and capsule opacification (D'Eliseo et al., 2003; Halili et al., 2014; Zhang et al., 2021). These beneficial effects may at least partially result from an increased magnitude and uniformity of the stress field within the capsule that reduces the mechanical perturbation caused by cataract surgery and implantation of a non-axisymmetric IOL. Indeed, we recently developed a 3-D finite element model of the lens capsule after cataract surgery with an implanted CTR and demonstrated that it induced a nearly uniform stress field in the remnant capsule, albeit with a lower magnitude than homeostatic (Berggren et al., 2021). An important limitation of this model is that it did not consider cell-mediated remodeling of the lens capsule over time after cataract surgery, which is known to dramatically affect its interaction with implants. Although we have previously reported an adaptive model of the lens capsule with a CCC using a growth (changes in mass) and remodeling (changes in microstructure) (G&R) framework, it was an axisymmetric (1-D) model that only included the anterior portion of the capsule without an implanted device (Pedrigi and Humphrey, 2011).

Therefore, herein, we extended our 3-D finite element (FE) model of the post-surgical lens capsule by coupling it to an adapted version of our previously reported G&R framework to assess the impact of implanting a CTR with the IOL on the evolving mechanics over 4 years after cataract surgery. Our FE-G&R models tracked changes to the constituents of the post-surgical capsule at each element, thus allowing for non-axisymmetric adaptations. To our knowledge, this is the first model of the post-surgical lens capsule that can predict implant efficacy over time after cataract surgery. Importantly, efficacy is defined in terms of a key patient outcome: the extent of fibrosis development. We found that implantation of a CTR with the IOL reduced thickening and stiffening of the lens capsule along the visual axis compared to when the IOL was implanted alone. This finding suggests that implantation of CTRs in the lens capsules of normal patients could reduce the incidence of PCO.

## 2 Materials and methods

### 2.1 Finite element modeling

All modeling, meshing, and analyses were performed in Abaqus CAE 2019. The lens capsule was meshed with a combination of 3-node shell elements and 4-node reduced integration shell elements (S4R) and the CTR and IOL were meshed with 8-node 3-D continuum elements (C3D8). The final mesh density of each component of the models was determined with a convergence test of the following metrics: for the lens capsule, when the displacement of the capsule pole differed by <1% with an increase in mesh density; for the CTR and IOL, when the displacement of the capsule equator differed by <1%. A description of the salient aspects of each model are given below.

#### 2.1.1 Model of the post-surgical lens capsule with IOL and CTR-IOL

**FIGURE 1:**
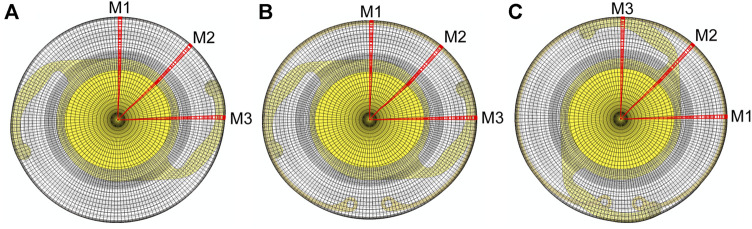
Images of the three FE models evaluated in this study showing the orientation of meridians used to report results. The three models are: **(A)** the post-surgical lens capsule with implanted IOL, **(B)** the post-surgical lens capsule with implanted CTR and IOL with the IOL haptics aligned horizontally (CTR-IOL Case 1), and **(C)** the post-surgical lens capsule with implanted CTR and IOL with the IOL haptics aligned vertically (CTR-IOL Case 2). Each model has three distinct meridians highlighted (M1, M2 and M3), with M1 perpendicular to the IOL haptics, M2 at an approximately 45° angle from the haptics, and M3 parallel to the IOL haptics.

The initial equatorial diameter and thickness profile of the post-surgical lens capsule were the same as used in our previous study (Ameku and Pedrigi, 2022). A 5 mm-diameter CCC was placed in the anterior portion. The unloaded geometry was modeled as a flattened circular membrane with a flat equatorial region and small gap between the anterior and posterior portions. This gap was set as the thickness of the implanted IOL because this is the primary determinant of the post-surgical capsule geometry (along with the zonules attached at the equator) due to contraction in the first weeks after surgery that causes the capsule to be in apposition to the implant. Thus, the initial state of the model represents the capsule ∼2 weeks after surgery (Hayashi et al., 2002; Pedrigi et al., 2009a). Two implants were considered, either an IOL alone (IOL model) or the combination of a CTR and IOL (CTR-IOL model). In line with our previous work (Pedrigi and Humphrey, 2011), we assumed the lens capsule was stress free prior to placement of the implanted IOL. The interaction between the post-surgical lens capsule and implanted IOL was modeled with separation allowed after contact, but the post-surgical lens capsule and CTR interaction was modeled with no separation after contact because it provided better stability. All other contact settings for the post-surgical model were set to match those of our previously reported native lens model (Ameku and Pedrigi, 2022). For the IOL simulation, the optic portion of the IOL was centered with respect to the lens capsule and held in place. The haptics were compressed with an applied traction to bring them within the capsule and then released to allow contact with the capsule equator. The lens capsule equator and IOL were restricted to in-plane motion and the lens capsule was not allowed to rotate (Berggren et al., 2021). For the CTR-IOL simulation, the IOL was modeled with the same approach as the simulation with the IOL alone, and the CTR was placed within the capsule similar to the IOL by compressing the loop ends radially and circumferentially with an applied traction and then releasing them to allow contact with the capsule equator. In this simulation, the lens capsule equator and CTR were restricted to in-plane motion, the top portion of the CTR was held to only allow radial motion, and the lens capsule was not allowed to rotate. No interaction between the CTR and IOL was considered because these implants are not designed for coupling within the capsular bag (i.e., they are implanted separately, one above or below the other; for simplicity, we just eliminated the interaction). All boundary conditions regarding the IOL were the same as described for the IOL model. Additionally, to determine whether or not the orientation of the implanted IOL with respect to the CTR affects the results of our model, two cases were considered: CTR-IOL Case 1 considered the IOL haptics aligned perpendicular to the CTR opening (horizontally from the front viewpoint) and CTR-IOL Case 2 considered the IOL haptics aligned with the CTR opening (vertically from the front viewpoint). Videos of each FE simulation are provided in Supplementary Videos S1–S3. Model outputs are provided along three meridians, referred to as M1, M2, and M3, for all models (Figure 1).

#### 2.1.2 Model outputs

For all finite element analyses, the primary readouts were the Cauchy stress tensor and the deformation gradient tensor, ***F***, at each increment of the total simulated time. Both metrics were determined with respect to the circumferential and meridional (principal) directions of the lens capsule in all simulations. These were determined to be the principal directions from our previous mechanical (inflation) testing of the lens capsule, wherein shear strain was negligible with respect to these directions (Heistand et al., 2005; Pedrigi et al., 2007).

### 2.2 Mechanical properties

#### 2.2.1 Lens capsule

We employed the Holzapfel hyperelastic constitutive model that was previously fit to inflation (Pedrigi et al., 2007) and uniaxial (Krag et al., 1997) mechanical testing data for the anterior lens capsule from human donors of comparable age (∼65 years) to that being modeled in this study (Berggren et al., 2021; Ameku and Pedrigi, 2022). The constitutive model in terms of Cauchy stress is given by

$$\sigma =F\frac{\partial W_{H}}{\partial F^{T}}-pI$$ (1)

where ***F*** is the deformation gradient tensor, *p* is the Lagrange multiplier used to enforce incompressibility, ***I*** is the identity tensor, and *W*_*H*_ is the Holzapfel strain energy function given by

$$W_{H}=C_{10}\left(I_{1}-3\right)+\frac{k_{1}}{k_{2}}\left\{\exp\left[k_{2}{\left(\kappa I_{1}+\left(1-3\kappa \right)I_{4}-1\right)}^{2}\right]-1\right\}$$ (2)

where *C*_*10*_ is the ground matrix stiffness, *k*_*1*_ is the stiffness of the fiber families, *k*_*2*_ is a dimensionless material parameter, *κ* signifies the in-plane distribution of the fibers, and *I*_*1*_ and *I*_*4*_ are the first and fourth invariants of the right Cauchy-Green tensor, respectively (note, *I*_*4*_ also contains the mean fiber distribution angle, γ). In our previous study (Berggren et al., 2021), we determined the values for these parameters that allowed accurate prediction of the regionally-varying anisotropic mechanical behavior exhibited by the anterior capsule during inflation testing. The final material parameters slightly changed over the meridian of the lens capsule in an element-to-element fashion owing to an increasingly stiffer circumferential direction and more compliant meridional direction from pole to equator. These regional variations in mechanical properties were implemented into the finite element (FE) model using a custom MATLAB script, as previously described (Berggren et al., 2021).

#### 2.2.2 Implanted CTR and IOL

The CTR model was based on a Morcher type 14 capsular tension ring with a maximum undeformed diameter of 12.3 mm and a deformed diameter of 10 mm that is uniform when the loop ends come nearly into contact (Menapace et al., 2000). The CTR was given a thickness of 0.2 mm based on previous models of capsular measuring rings made by our group (Berggren et al., 2021). The IOL model was based on a single-piece Alcon AcrySof monofocal IOL made of hydrophobic acrylic with an overall length of 13 mm, optic diameter of 6 mm, and thickness of 0.2 mm to match the thickness of the CTR (Nejima et al., 2006; Werner et al., 2018). Since both implants undergo small strains (<1%), we used a linear elastic constitutive model, with a Young’s modulus of 3.2 GPa and a Poisson’s ratio of 0.37 assigned to the CTR, and a Young’s modulus of 12 MPa and Poisson’s ratio of 0.37 assigned to the IOL (Berggren et al., 2021).

### 2.3 Growth and remodeling

Our prior growth and remodeling (G&R) work of the lens capsule (Pedrigi and Humphrey, 2011), which was based on studies in vascular mechanics (Baek et al., 2006; Valentin et al., 2013; Latorre and Humphrey, 2018), has demonstrated that a constrained mixture model can effectively capture cell-mediated tissue adaptations driven by altered mechanics. Herein, we used this framework to simulate G&R of the entire lens capsule with the aforementioned implants after cataract surgery in weekly increments over 4 years.

#### 2.3.1 Framework

This framework tracks changes in the deposition and removal of each of the primary load-bearing constituents of the lens capsule, denoted *k*, and computes the associated strain energy at each G&R time (following a perturbation at time 0) using

$$W_{F}^{k}\left(s\right)=\frac{M^{k}\left(0\right)}{M\left(s\right)}Q^{k}\left(s\right){\hat{W}}_{F}^{k}\left(\lambda_{n\left(0\right)}^{k}\left(s\right)\right)+\int_{0}^{s}\frac{m^{k}\left(\tau \right)}{M\left(s\right)}q^{k}\left(s,\tau \right){\hat{W}}_{F}^{k}\left(\lambda_{n\left(\tau \right)}^{k}\left(s\right)\right)d\tau ,$$ (3)

where *M(s)* is the total mass (note, $M\left(s\right)=\sum M^{k}\left(s\right)$ ) at the current G&R time *s*, *M* ^*k*^ is the mass of constituent *k* (referred to by a superscript *k* in all instances), *M* ^*k*^(0) is the mass density in the homeostatic state, *Q* ^*k*^(s) is the fraction of mass produced in the homeostatic state that survives to the current time *s*, *m* ^*k*^ is the rate of mass production at G&R time $\tau \in \left[0,s\right]$ , *q* ^*k*^ is the fraction of mass that was produced at time *τ* that survives to time *s*, and *W* ^*k*^ is the strain energy of constituent *k* summed over all cohorts (each cohort deposited at a specific G&R time *τ*). We employed a Fung-type exponential form of the strain energy function for each constituent given by

$${\hat{W}}_{F}^{k}\left(\lambda_{n\left(\tau \right)}^{k}\left(s\right)\right)=c^{k}\left\{\exp\left[c_{1}^{k}{\left({\left(\lambda_{n\left(\tau \right)}^{k}\right)}^{2}-1\right)}^{2}\right]-1\right\},$$ (4)

where *c* is a measure of overall constituent stiffness, *c*_*1*_ is a non-dimensional stiffness parameter, and $\lambda_{n}^{k}$ is the stretch experienced by a cohort of constituent *k* deposited at G&R time *τ*. This stretch is computed via

$$\lambda_{n\left(\tau \right)}^{k}\left(s\right)=G_{h}^{k}\frac{\lambda^{k}\left(s\right)}{\lambda^{k}\left(\tau \right)},$$ (5)

where $G_{h}^{k}$ denotes the deposition stretch at which the constituent is incorporated within the extant capsule matrix and *λ* ^*k*^(*τ*) is the stretch of the gross capsule in the direction of constituent *k* at G&R time *τ*, given by

$$\lambda^{k}\left(\tau \right)=\sqrt{{\left(\lambda_{1}\cos\left(\alpha^{k}\right)\right)}^{2}+{\left(\lambda_{2}\sin\left(\alpha^{k}\right)\right)}^{2}}$$ (6)

where *λ*_*1*_ and *λ*_*2*_ are the principal stretches in the circumferential and meridional directions, respectively, and *α* is the angle of constituent *k* relative to the circumferential direction.

The primary load-bearing constituent of the native lens capsule is type IV collagen, whereas after cataract surgery non-native fibrillar collagen (types I, III, and V) is also deposited. Thus, our G&R model considered two families of native collagen (type IV) and two families of fibrillar collagen. Rates of mass production and removal of these constituents were determined based on changes in stress from homeostatic using

$$m^{k}\left(\tau \right)=m_{o}^{k}\left(1+K_{P}^{k}\Delta \sigma \left(\tau \right)\right)$$ (7)

and

$$q^{k}\left(s,\tau \right)=\exp\left(-q_{o}^{k}\left[1+K_{R}^{k}\Delta \sigma \left(s\right)\right](\mathrm{s-\tau})\right),$$ (8)

respectively, where *m*_*0*_ is the basal rate of mass production, *q*_*o*_ is the basal rate of mass removal based on an estimate from clinical observations of lens capsule turnover (Pedrigi and Humphrey, 2011), and *K*_*P*_ and *K*_*R*_ are non-dimensional gain-type parameters that amplify changes in the production and removal rates, respectively, based on the magnitude of change in the principal stresses from homeostatic of the gross lens capsule, given by

$$\Delta \sigma =\left|\frac{\sigma_{11}+\sigma_{22}}{{\left(\sigma_{11}\right)}_{o}+{\left(\sigma_{22}\right)}_{o}}-1\right|$$ (9)

where *o* denotes original homeostatic, which were obtained from a finite element model of the native lens (Berggren et al., 2021; Ameku and Pedrigi, 2022), and *11* and *22* indicate the circumferential and meridional directions, respectively. These gain parameters were optimized to achieve a desired increase in thickness at the CCC edge based on previously reported anterior capsule opacification (ACO) scores for human cadaver lens capsules that had undergone cataract surgery more than 3 years before the time of death (Maddula et al., 2011). We also calibrated the gain-type parameters of our model to reasonably approximate the increased stiffness reported in our previous study (Pedrigi et al., 2009b; Pedrigi and Humphrey, 2011).

#### 2.3.2 Coupling the G&R framework to the FE model of the post-surgical lens capsule

All simulations used MATLAB as a shell to run both the G&R framework, which is programmed in MATLAB, and the FE models in Abaqus. Because the FE models were non-axisymmetric, mass deposition and removal were tracked at each element over all G&R times after cataract surgery. An FE model of the native lens capsule and fibers was used to determine the homeostatic stress and stretch fields of the capsule (Berggren et al., 2021). The FE models of the post-surgical lens capsule with implanted device (either IOL or CTR-IOL) provided the stress and stretch fields at each G&R time *s*. The FE models employed the Holzapfel constitutive model, whereas changes in the rates of mass production and removal of both native and fibrillar collagen were accounted for using the Fung-type constitutive model. Parameters for the Fung-type model were obtained at *s* < 0 when the lens capsule was entirely composed of type IV collagen (i.e., before G&R) by using a nonlinear regression to fit biaxial Cauchy stress-stretch data in the circumferential and meridional directions generated from the Holzapfel model via

$$\sigma_{11}=\lambda_{1}\frac{\partial W_{H}}{\partial \lambda_{1}}-\lambda_{3}\frac{\partial W_{H}}{\partial \lambda_{3}}\equiv \lambda_{1}\sum_{k=1}^{4}\frac{\partial W_{F}^{k}}{\partial \lambda_{1}}$$ (10)

$$\sigma_{22}=\lambda_{2}\frac{\partial W_{H}}{\partial \lambda_{2}}-\lambda_{3}\frac{\partial W_{H}}{\partial \lambda_{3}}\equiv \lambda_{2}\sum_{k=1}^{4}\frac{\partial W_{F}^{k}}{\partial \lambda_{2}}.$$ (11)

**FIGURE 2:**
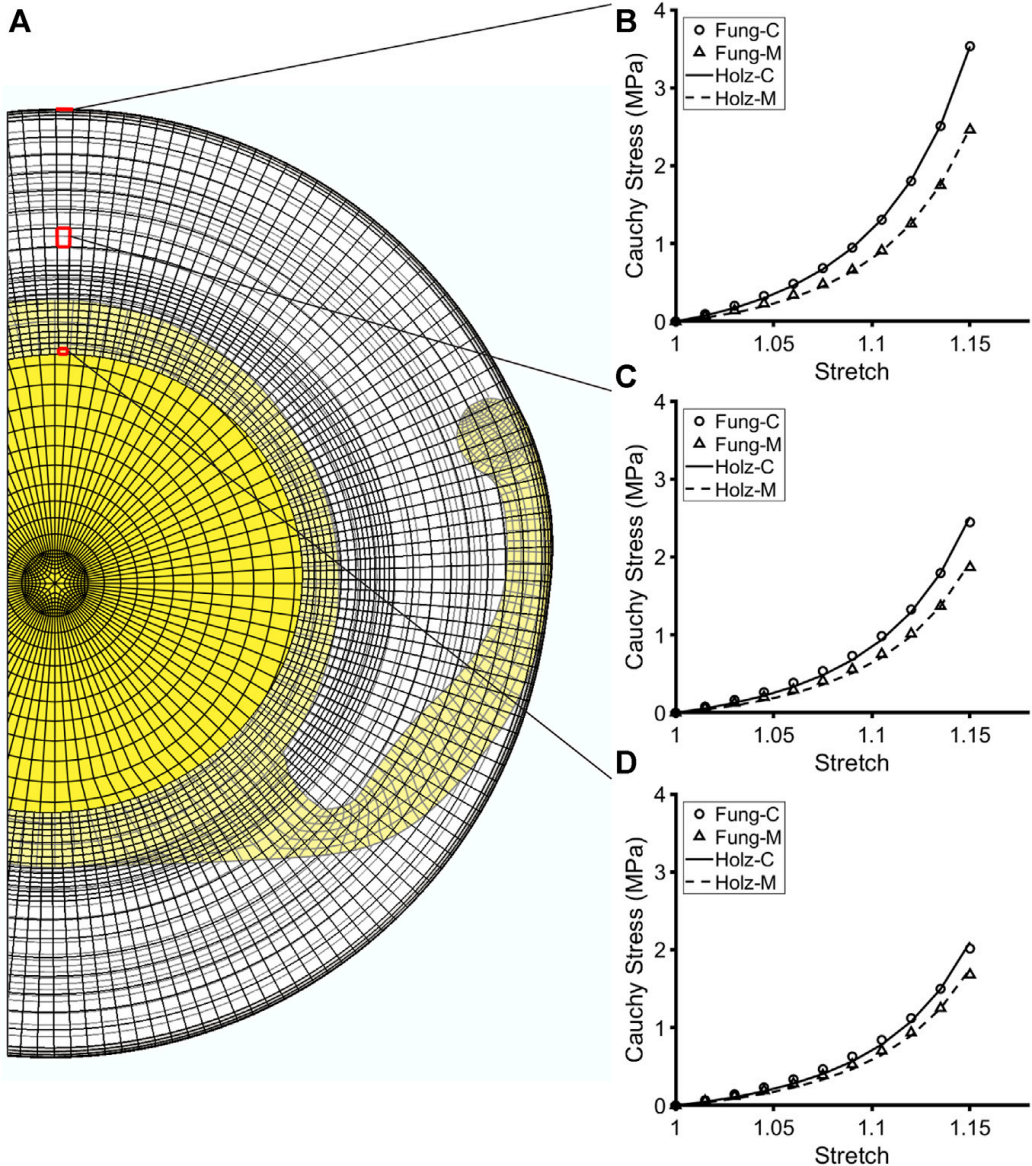
Fit of the Holzapfel constitutive model to simulated biaxial mechanical data from the Fung-type model used to compute G&R of the post-surgical capsule immediately after cataract surgery. **(A)** Cut view of the post-surgical FE model with an implanted IOL. **(B–D)** Representative elements highlighted at the CCC edge, anterior midpoint, and equator, showing the anisotropic mechanical behavior of the capsule at each element. Plots illustrate the goodness of fit of the Holzapfel constitutive model (through the parameter *k*_*1*_) to the biaxial mechanical data generated from the Fung-type model at each respective element, thus validating the approach.

**FIGURE 3:**
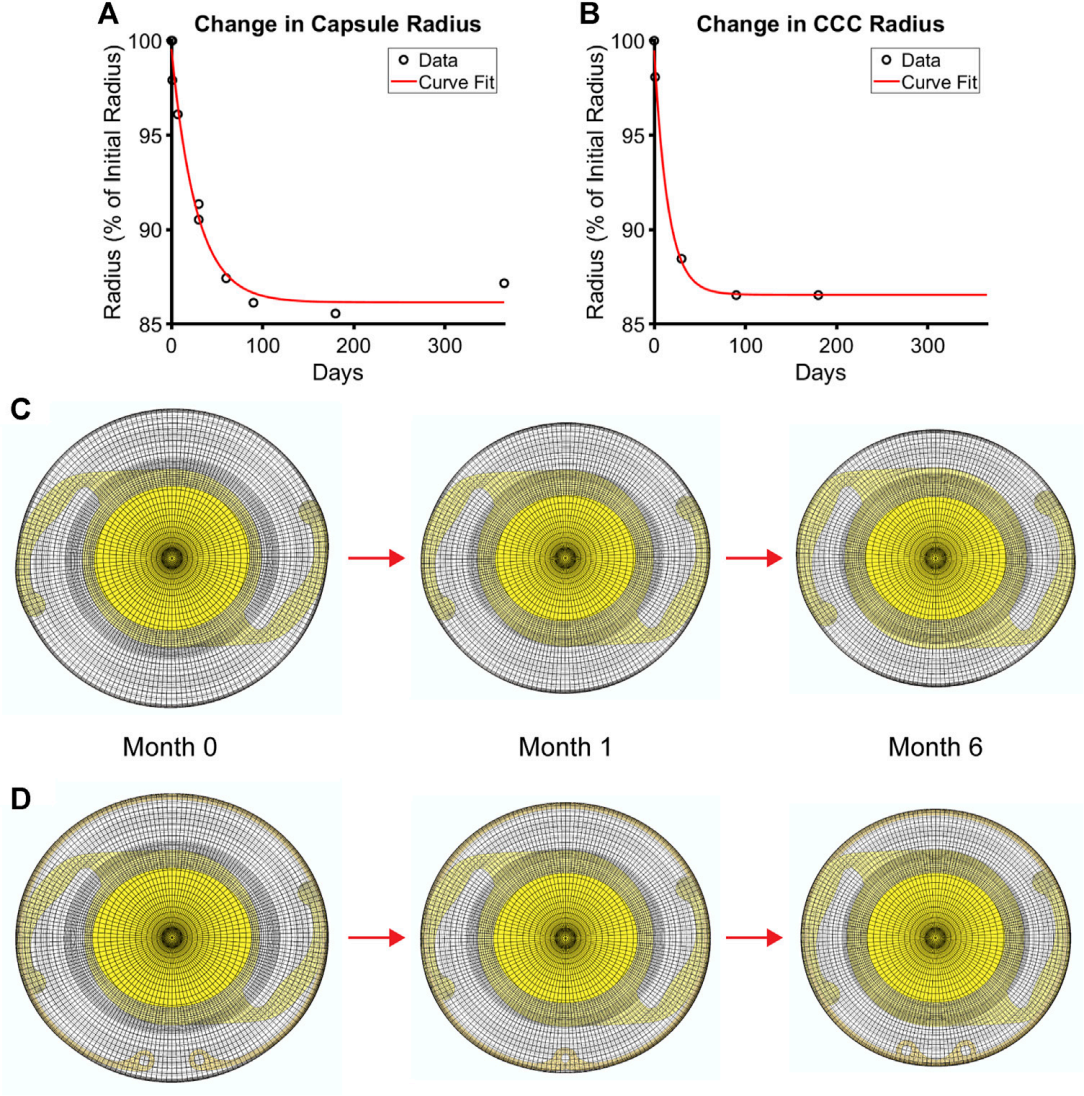
Contraction of the post-surgical lens capsule models over time after cataract surgery. Empirical data and the associated fit of changes in **(A)** capsule equatorial radius and **(B)** CCC radius. FE models of the **(C)** post-surgical capsule with implanted IOL and **(D)** post-surgical capsule with implanted CTR and IOL each at the simulated G&R times of 0 (i.e., immediately after surgery), 1, and 6 months after surgery when full contraction is reached.

The material parameters for fibrillar collagen were estimated by assuming that *c* ^*k*^ is an order of magnitude larger than that for type IV collagen and the other parameters, *c*_*1*_^*k*^ and *α* ^*k*^ *,* were held constant for both types of collagen (Pedrigi and Humphrey, 2011). At every G&R time *s* ≥ 0, this process was done in reverse, wherein the Fung-type constitutive model was used to generate biaxial Cauchy stress-stretch data that were fit using the Holzapfel model through the stiffness parameter *k*_*1*_. Because the two constitutive models described the lens capsule mechanical behavior very similarly, resultant fits were excellent (Figure 2). Using this approach, *k*_*1*_ represented the overall stiffness of the lens capsule constituents, including contributions from both type IV collagen and type I collagen (note that a higher deposition rate of type IV collagen compared to removal only changes the thickness of the capsule, not the stiffness, while that for non-native type I collagen changes both thickness and stiffness). In addition, we did not consider changes in the anisotropy of the capsule over time because the degree of anisotropy in the native lens capsule predicted by the Holzapfel model is modest and there are no data characterizing changes after cataract surgery. As a result, we did not consider changes to the other parameters of the Holzapfel model. Once changes in stiffness (*k*_*1*_ parameter of the Holzapfel model) and thickness (based on changes in mass) were determined from the G&R framework at each element, they were passed to the FE model of the post-surgical capsule with implant to run the next G&R time step of the loop. Contraction of the capsule equator and CCC were also prescribed in the FE model by incrementally reducing each diameter over the first 6 months of simulation time based on empirical data from patients (Tehrani et al., 2003) (Figure 3).

Finally, as the lens capsule remodels after cataract surgery, the lens epithelial cells migrate to the posterior capsule, which is natively acellular, and over time errant behaviors (e.g., contraction and matrix synthesis) cause the development of posterior capsule opacification. This complication of cataract surgery can take months to years to unfold, depending on the IOL geometry (square versus rounded edge optic). Since a square-edged IOL optic delays the migration of cells to the posterior capsule, we incorporated this feature into the remodeling process using a custom MATLAB program. Specifically, we assumed an immediate cellular migration up to the posterior IOL optic edge and then imposed a 1 year delay before allowing migration to the midpoint of the IOL optic (∼1.5 mm from the posterior pole) based on previously reported ACO and PCO scores (Maddula et al., 2011). As a result, in the first year of simulation, changes to the constituents only occurred in the remnant anterior capsule and posterior capsule up to the edge of the visual axis; thereafter, we also simulated changes from the edge of the visual axis to 1.5 mm from the posterior pole to consider the development of peripheral PCO (pathology of human cadaver eyes with implanted square-edged IOLs showed ACO and PCO in the peripheral portion of the posterior capsule, but not PCO in the central portion (Maddula et al., 2011), so remodeling in this portion was not considered).

## 3 Results

**FIGURE 4:**
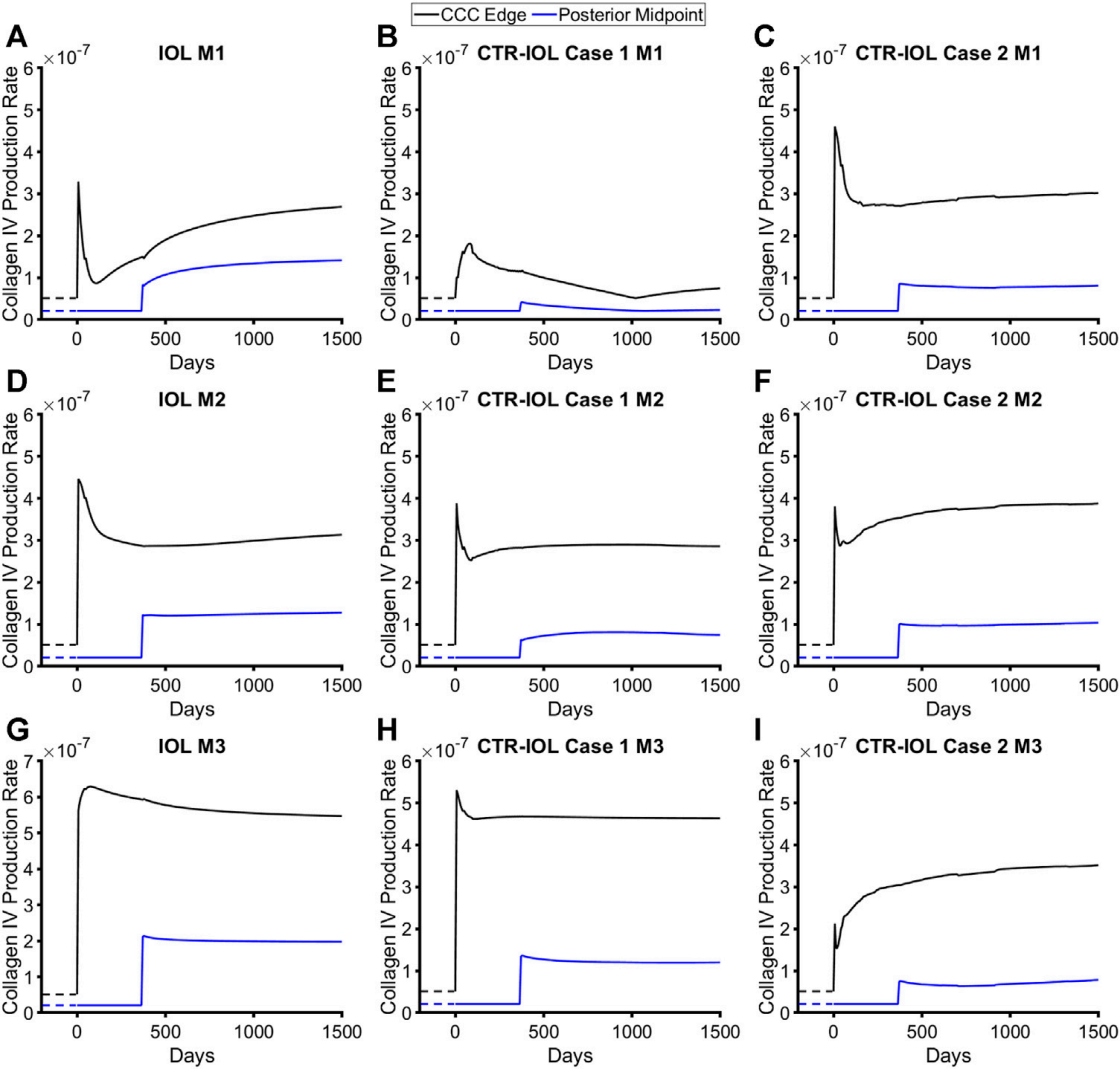
Post-surgical lens capsule mass production rates of type IV collagen for the FE-G&R models over time after cataract surgery. The three models are: **(A,D,G)** the post-surgical lens capsule with implanted IOL, **(B,E,H)** the post-surgical lens capsule with implanted CTR and IOL where the IOL haptics are aligned horizontally (Case 1), and **(C,F,I)** the post-surgical lens capsule with implanted CTR and IOL where the IOL haptics are aligned vertically (Case 2). Mass production rates are shown at the CCC edge and posterior midpoint along M1 (top row), M2 (middle row), and M3 (bottom row). Homeostatic values are shown for comparison (dashed lines).

Our FE-G&R model predicted cell-mediated changes in constituent mass, which led to changes in thickness and stiffness, of the lens capsule over 4 years after cataract surgery based on changes in stress from homeostatic at each element within each model. We calibrated our growth parameters to achieve predictions of capsule thickening and stiffening based on previously reported empirical data (Pedrigi et al., 2009b; Maddula et al., 2011). Final parameters were [*K*_P_^*IV*^, *K*_R_^*IV*^] = [7.5, 1] for native (type IV) collagen and [*K*_P_^*I*^, *K*_R_^*I*^] = [2, 1] for non-native fibrillar (type I) collagen. These growth parameters were then held constant for all models to allow comparisons. All models predicted dramatic increases in the production rates of native and fibrillar collagen (Figure 4; Supplementary Figure S1).

The trends over time were identical between the two constituents (note, there are four constituents in the model, two each of the two types of collagen) and qualitatively similar between the implants with initial spikes in mass production that mostly reduced over time (a few exhibited increases), but remained above pre-surgery homeostatic values. The equatorial regions of the capsule in contact with the implant saw by far the highest rates of mass production (Supplementary Figure S2). Because the CTR contacts the lens capsule over nearly the entire circumference, models with this device exhibited higher mass production over a larger area of the equator than models with the IOL alone, where only the region of the capsule contacting the haptics exhibited similar levels of mass production. However, along the visual axis, which we defined as the central region of the post-surgical lens capsule (remnant anterior and posterior) that covered the 6 mm diameter IOL optic, models with the CTR predicted overall lower amounts of mass production than the model with the IOL alone. Specifically, the CTR-IOL Case 1 model showed significantly smaller increases in the rates of mass production compared to the IOL model along M1 (Figures 4A, B) and slightly smaller increases in the direction of M2 (Figures 4D, E) and M3 (Figures 4G, H) in both the anterior and posterior portions of the post-surgical capsule. The CTR-IOL Case 2 model showed an increase in the rates of mass production in the anterior portion of the capsule compared to the IOL model along M1, but decreases along M2 and M3. Similar to the CTR-IOL Case 1 model, mass production in the Case 2 model was uniformly decreased in the posterior capsule (Figures 4C, F, I). This model also exhibited more uniform mass production along all meridians compared to the other models.

**FIGURE 5:**
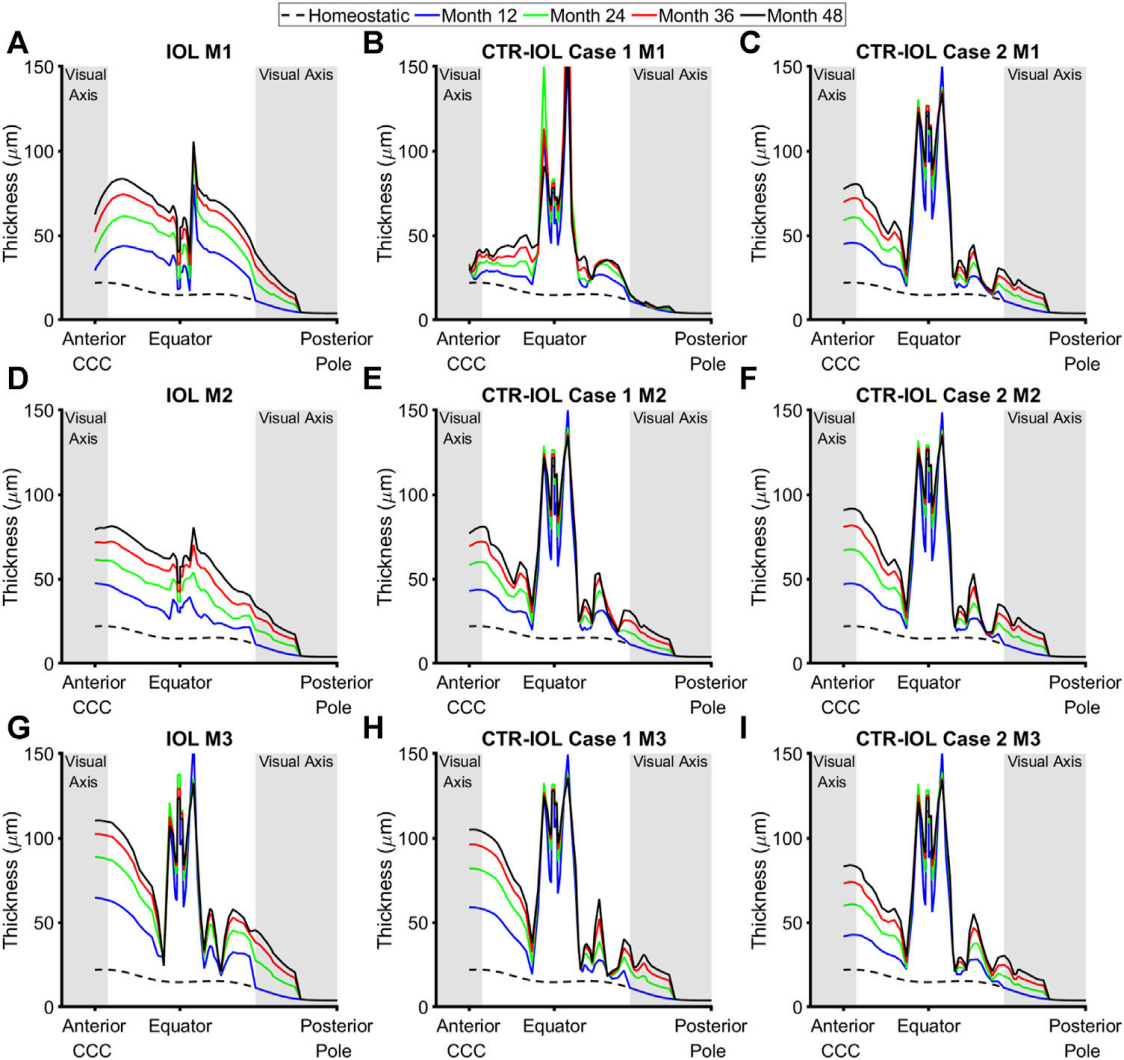
Post-surgical lens capsule thickness of the FE-G&R models at select simulation times up to the maximum of 4 years after cataract surgery. The three models are: **(A,D,G)** the post-surgical lens capsule with implanted IOL, **(B,E,H)** the post-surgical lens capsule with implanted CTR and IOL where the IOL haptics are aligned horizontally (Case 1), and **(C,F,I)** the post-surgical lens capsule with implanted CTR and IOL where the IOL haptics are aligned vertically (Case 2). Thickness at each time is shown from the CCC edge to the posterior pole along M1 (top row), M2 (middle row), and M3 (bottom row). The regions of the anterior and posterior visual axes are highlighted in grey. Homeostatic values are shown for comparison (dashed black line).

In line with predicted rates of mass production, implantation of a CTR led to more uniformly increased thickening around the equator of the post-surgical capsule, but lower thickening along the visual axis compared to implantation of an IOL alone. Specifically, the CTR-IOL Case 1 model predicted a mean anterior thickening along the visual axis at the end of 4 years of 14 μm versus 49 μm in the IOL model in the direction of Meridian 1 (M1) (Figures 5A, B), 57 μm versus 58 μm (M2) (Figures 5D, E), and 83 μm versus 88 μm (M3) (Figures 5G, H **)**. While the CTR-IOL Case 2 model predicted a much more uniform thickening between meridians with mostly higher values compared to the CTR-IOL Case 1 model of 57 μm (M1), 69 μm (M2), and 62 μm (M3) (Figures 5C, F, I). The posterior portion of the post-surgical lens capsule exhibited even more dramatic differences in thickness along the visual axis between both CTR-IOL models versus the IOL model. Here, the CTR-IOL Case 1 model predicted a mean thickening of 1 μm versus 13 μm in the IOL model in the direction of Meridian 1 (M1) (Figures 5A, B), 9 μm versus 12 μm (M2) (Figures 5D, E), and 12 μm versus 18 μm (M3) (Figures 5G, H). The CTR-IOL Case 2 model predicted a mean thickening of 9 μm (M1), 11 μm (M2), and 9 μm (M3) (Figures 5C, F, I).

**FIGURE 6:**
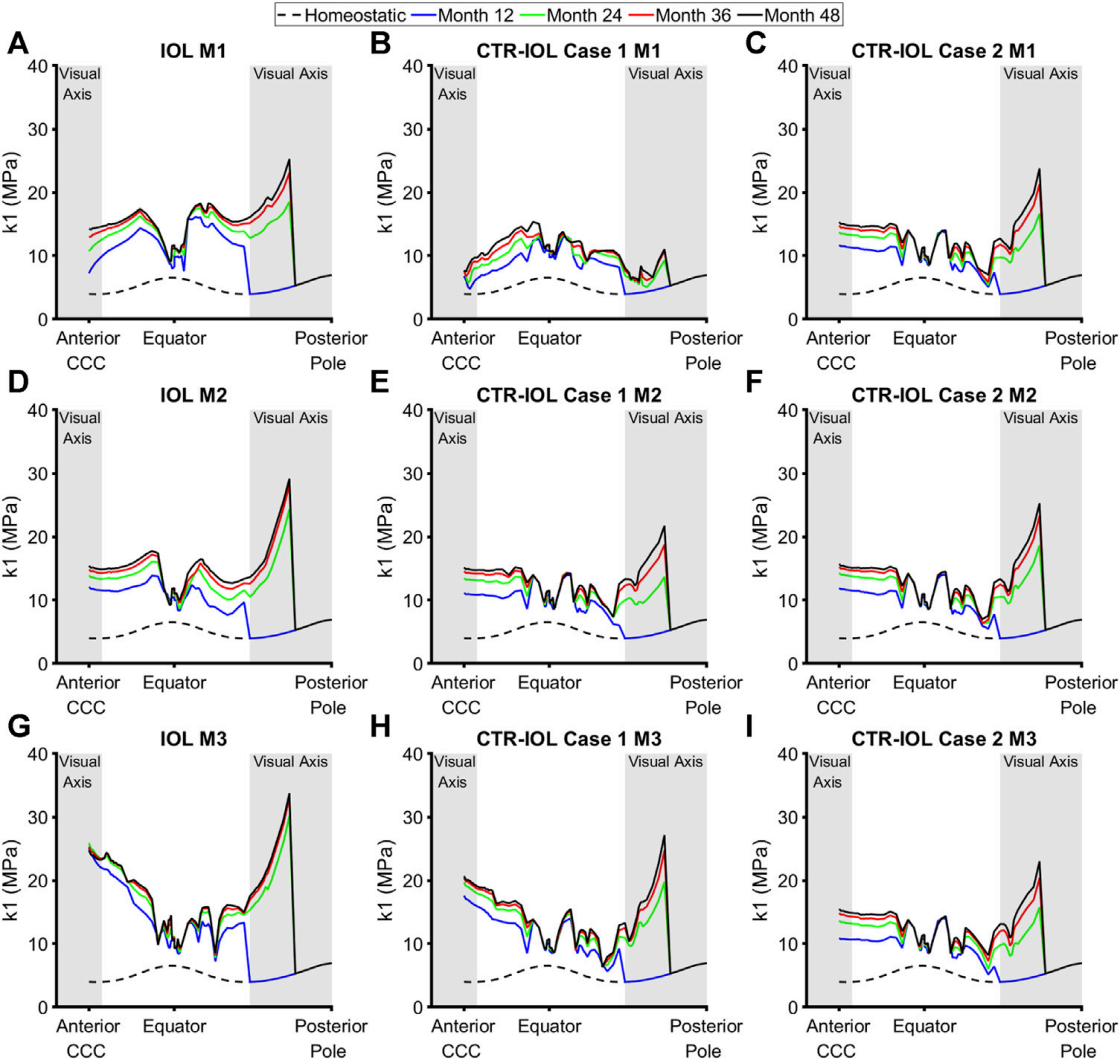
Post-surgical lens capsule stiffness (*k*_*1*_ parameter of the Holzapfel model) of the FE-G&R models at select simulation times up to the maximum of 4 years after cataract surgery. The three models are: **(A,D,G)** the post-surgical lens capsule with implanted IOL, **(B,E,H)** the post-surgical lens capsule with implanted CTR and IOL where the IOL haptics are aligned horizontally (Case 1), and **(C,F,I)** the post-surgical lens capsule with implanted CTR and IOL where the IOL haptics are aligned vertically (Case 2). Stiffness at each time is shown from the CCC edge to the posterior pole along M1 (top row), M2 (middle row), and M3 (bottom row). The regions of the anterior and posterior visual axes are highlighted in grey. Homeostatic values are shown for comparison (dashed black line).

Stiffness (the *k*_*1*_ parameter of the Holzapfel model) followed a similar trend as thickness, but with even larger differences between implanted devices. In the anterior visual axis region of the post-surgical lens capsule, the CTR-IOL Case 1 model predicted a mean stiffening at the end of 4 years of 4.78 MPa versus 10.43 MPa in the IOL model in the direction of Meridian 1 (M1) (Figures 6A, B), 10.91 MPa versus 11.10 MPa (M2) (Figures 6D, E), and 15.86 MPa versus 19.81 MPa (M3) (Figures 6G, H). In line with the thickness results, the CTR-IOL Case 2 model also predicted a much more uniform stiffening between meridians with mean values of 10.99 MPa along M1, 11.38 MPa along M2, and 11.14 MPa along M3 (Figures 6C, F, I). In the posterior visual axis region, the CTR-IOL Case 1 model predicted a mean stiffening of 2.21 MPa versus 9.98 MPa in the IOL model in the direction of Meridian 1 (M1) (Figures 6A, B), 7.49 MPa versus 9.50 MPa (M2) (Figures 6D, E), and 8.08 MPa versus 12.36 MPa (M3) (Figures 6G, H). The CTR-IOL Case 2 model predicted a mean stiffening in the posterior visual axis region of 7.36 MPa (M1), 7.97 MPa (M2), and 7.26 MPa (M3) (Figures 6C, F, I).

Since changes in stress (Δσ) drove predicted changes in thickness and stiffness, models with the CTR generally predicted overall lower amounts of change in stress than the model with the IOL alone (Supplementary Figure S3). We also quantified changes in thickness, stiffness, and stress over every meridian of the anterior and posterior visual axis regions of the post-surgical lens capsule, which showed similar trends to results presented for the individual meridians (Supplementary Figure S4).

## 4 Discussion

In this study, we developed an FE-G&R model to evaluate how implantation of a CTR in combination with an IOL (using two different IOL orientations relative to the CTR) influences mechanical adaptations of the lens capsule over 4 years after cataract surgery compared to when the IOL is implanted alone. All models predicted significantly increased thickening and stiffening at the point of implant contact with the capsule equator, but the models with the CTR predicted these increases more uniformly around the equator due to the increased contact area. However, fibrotic remodeling at the capsule equator would not affect patient vision because it is away from the visual axis, which we defined as the anterior and posterior portions of the capsule that are over the 6 mm-diameter IOL optic. Along the visual axis, models with the CTR predicted a reduced mechanical perturbation of the lens capsule and, in turn, reduced thickening and stiffening compared to the model of the IOL alone. Orientation of the IOL haptics relative to the CTR opening showed only a modest impact on the average results wherein the perpendicular orientation (Case 1) exhibited slightly lower mean values of thickening and stiffening. On the other hand, the parallel orientation (Case 2) exhibited much more uniform remodeling. Overall, these reductions in fibrotic remodeling along the visual axis with implantation of a CTR were particularly noticeable in the posterior portion of the capsule, suggesting that it would lead to better patient outcomes in terms of reduced severity of PCO and associated visual disturbances. This prediction aligns with a recent meta-analysis of eight studies that acquired patient data from 379 eyes implanted with a CTR in combination with an IOL and 333 eyes implanted with an IOL alone (Zhang et al., 2021). They found that eyes implanted with a CTR exhibited a significant reduction in the rate of posterior capsulotomy (a laser-based surgery that serves as the primary treatment for PCO) and PCO score compared to those implanted with the IOL alone (odds ratio of 0.24 and standardized mean difference of −1.40, respectively).

While many studies have modeled the native lens (Burd et al., 2002; Hermans et al., 2006; Reilly, 2014; Burd and Wilde, 2016; David et al., 2017; Cabeza-Gil et al., 2021; Knaus et al., 2021; Knaus et al., 2023), only a few studies have modeled the lens capsule after cataract surgery (Pedrigi and Humphrey, 2011; Berggren et al., 2021; Ameku and Pedrigi, 2022; Cabeza-Gil and Calvo, 2022). Moreover, to our knowledge, this is the first FE-G&R model of the lens capsule in any context. Our G&R framework tracked changes in the deposition and removal of the primary load-bearing constituents of the post-surgical lens capsule, native type IV collagen and non-native (fibrillar) type I collagen, at each element based on changes in the principal stresses relative to homeostatic. To ensure that predicted changes in thickness of the post-surgical capsule were reasonable, we calibrated the type IV collagen growth parameters of the FE-G&R model of the IOL alone to semi-quantitative data reported from a study of fibrosis rates in 157 cadaver eyes from patients previously implanted with three-piece silicone IOLs with square edges that reported maximum thickness increases (at greater than 3 years post-operation) of ∼90 µm (Maddula et al., 2011) (which matches our maximum predicted thickening for the anterior capsule of 90 µm). Similarly, changes in stiffness were calibrated via the type I collagen growth parameters of this model to our previous results from uniaxial mechanical testing of post-surgical capsules demonstrating a ∼4-fold increase in stiffness around the CCC edge (Pedrigi et al., 2009b) (compared to the 3.7-fold increase predicted by our model). These growth parameters were then held constant across all models to allow comparisons.

Our G&R framework used a constrained mixture approach that allowed the native and fibrillar collagen constituents to have different mechanical properties and rates of turnover, but also constrained these constituents to move together within the mixture (i.e., the lens capsule at each material point). We assumed that cell-mediated changes in the mass of each constituent were driven by changes in stress within the post-surgical lens capsule for three primary reasons. First, fibrotic lens epithelial cell behaviors appear to last for years after cataract surgery (Marcantonio et al., 2000), which is long after the inflammatory response to the procedure has subsided (∼1 month), leaving the permanently altered mechanical environment of the capsule as the most obvious driver. Second, we previously demonstrated that the native anterior lens capsule exhibits a nearly homogeneous stress field that is significantly perturbed during cataract surgery (Pedrigi et al., 2007; Berggren et al., 2021). Third, epithelial cells, including lens epithelial cells, have been shown to be highly mechanosensitive (Kumar et al., 2019; Gao et al., 2022). These points align with numerous studies demonstrating that most cells are mechanosensitive and seek to maintain a homeostatic mechanical environment that, when perturbed, promotes pathologic cell behaviors (Humphrey, 2008; Nims et al., 2022). Many of these mechanobiology studies have focused on the vasculature where the G&R framework used herein was developed and extensively validated through studies of arteries in various applications, including the presence of hypertension (Latorre and Humphrey, 2018), altered blood flow (Karsaj et al., 2010), aneurysms (Baek et al., 2006), and external support (Ramachandra et al., 2020).

The FE-G&R model reported herein leverages years of previous work by us and others. We have previously characterized the mechanics of the native (Heistand et al., 2005; Pedrigi et al., 2007) and post-surgical (Heistand et al., 2006; Pedrigi et al., 2009a; Pedrigi et al., 2009b) lens capsule. We have also previously developed computational models of the lens capsule (Pedrigi et al., 2007; Pedrigi and Humphrey, 2011; David et al., 2017; Berggren et al., 2021; Ameku and Pedrigi, 2022). The current work directly builds on two of these computational studies. First, we previously developed a 1-D G&R model of the anterior portion of the lens capsule with central hole, but no implanted device (Pedrigi and Humphrey, 2011). We demonstrated an ability to calibrate the growth parameters of the model, which were driven by altered stress, to salient mechanical and biological data of the post-surgical lens capsule to predict pathological remodeling over time after surgery. We used an adaptation of this framework herein. Second, we previously developed an FE model of the entire post-surgical lens capsule with implanted IOL and, separately, CTR (Berggren et al., 2021). This model was calibrated to salient mechanical testing (inflation and uniaxial) data and validated. We reported changes to the stress field from homeostatic immediately after surgery, but not over time as there was no growth component to the model. Our study herein combined this FE model with our G&R model to report for the first time: (1) altered mechanics of the entire lens capsule over time after cataract surgery, including changes in thickness, stiffness, and stress; (2) the influence of different implants on predicted evolving lens capsule mechanics and associated fibrosis (ACO and PCO); and (3) implantation of an IOL plus CTR versus IOL alone reduces ACO and PCO. Importantly, this latter prediction qualitatively aligned with patient data showing a similar clinical outcome (Zhang et al., 2021).

There are several limitations of the study to consider. First, we assumed that the stress-free configuration of the capsule was circular due to zonular support, but did not consider the traction imposed by the zonules at the equator of the post-surgical capsule as it contracts and likely comes into tension with the zonules over time. However, this traction would only occur towards the end of the contraction period of the post-surgical capsule (based on previously reported initial diameters for the native lens, ciliary muscle, and post-surgical capsule; see (Strenk et al., 1999; Tehrani et al., 2003; Strenk et al., 2006)), which suggests that it would be small, and, at least in some regions of the capsule, the implant is pushing out against the capsule to disengage the zonules and nullify the traction; thus, it may be insignificant. This zonular traction has also never been measured or estimated. Second, we assumed that the circumferential and meridional directions of the capsule remain principal over remodeling time after cataract surgery. It is possible that the directions of the principal stresses change, which would alter the directions of new fiber deposition. However, the principal directions of a flat orthotropic membrane with a central hole subjected to a *uniform* radial traction are also circumferential and meridional (David and Humphrey, 2004), the non-axisymmetric nature of the loading due to the IOL is localized to only those portions of the capsule in line with the haptics (Berggren et al., 2021), and the degree of material anisotropy of the lens capsule is modest which means that small changes in fiber orientations would not be expected to significantly alter the material symmetry or mechanical behavior. Thus, while adding this complexity is something to consider for future work, we do not expect it to alter model predictions. Third, our model assumed cell migration to the two regions of the posterior capsule where remodeling occurred in two instantaneous steps, one to the IOL optic edge and the next to the mid-periphery of the posterior capsule (with a 1-year delay between them to account for the barrier effect of the sharp-edged IOL optic), thus ignoring continuous migration over time. While this limitation is likely to have only a small effect on predictions of long-term remodeling and little quantitative data exist to better characterize this phenomenon, it is an improvement that can be considered as more data become available. Fourth, there is little data quantifying microstructural changes to the lens capsule over time after surgery. We calibrated our growth parameters to semi-quantitative data from a report that characterized capsule fibrosis in 157 cadaver lenses of patients who had undergone surgery greater than 3 years previously (Maddula et al., 2011), but this study used a categorical scoring system of thickness ranges based on the maximum amount of fibrotic thickening present in the anterior portion of the capsules. There is a need for more detailed histological data that better quantifies capsule thickness and individual constituents spatially (over the capsule) and temporally (after cataract surgery).

## 5 Conclusion

Our FE-G&R models predicted that implantation of a CTR with the IOL into the lens capsule after cataract surgery reduced the mechanical perturbation, thickening, and stiffening along the visual axis in both the remnant anterior and posterior portions compared to implantation of an IOL alone. This finding aligns with patient studies and suggests that using this approach during routine cataract surgery would reduce visually debilitating ACO and PCO in all patients, not just those suffering from zonular weakness or dehiscence. To our knowledge, this is the first study to use modeling to demonstrate that the mechanical interactions of the implant and lens capsule play a significant role in determining evolving capsule mechanics and associated fibrosis over time after cataract surgery. It is also the first study to use modeling to predict the efficacy of any implant in terms of the extent of capsule fibrosis (i.e., ACO and PCO) development, which is the most significant complication of cataract surgery. Our work demonstrates that use of such modeling techniques has substantial potential to aid in the design of better surgical strategies and implants.

## Data Availability

The original contributions presented in the study are included in the article/Supplementary Material, further inquiries can be directed to the corresponding author.
