# Supplementary material for: Implantation of a capsular tension ring during cataract surgery attenuates predicted remodeling of the post-surgical lens capsule along the visual axis
Source: Front Bioeng Biotechnol. 2024 Jan 19;11:1300830. doi: 10.3389/fbioe.2023.1300830 (PMC10834774; doi:10.3389/fbioe.2023.1300830)
Supplement: Supplementary file 2 [file DataSheet4.PDF]

ELECTRONIC SUPPLEMENTARY MATERIAL

Implantation of a capsular tension ring during cataract surgery attenuates predicted remodeling of the post-surgical lens capsule along the visual axis

Kurt A. Ameku, Caleb C. Berggren, and Ryan M. Pedrigi

Department of Mechanical and Materials Engineering, University of Nebraska-Lincoln

Correspondence:

Ryan M. Pedrigi, Ph.D.

Mechanical and Materials Engineering

University of Nebraska-Lincoln

Nebraska Hall

P.0. Box 880526

Lincoln, NE 68588-0526

P: (402) 472-2732

F: (402) 472-8292

E: rpedrigi@unl.edu

SUPPLEMENTARY FIGURES

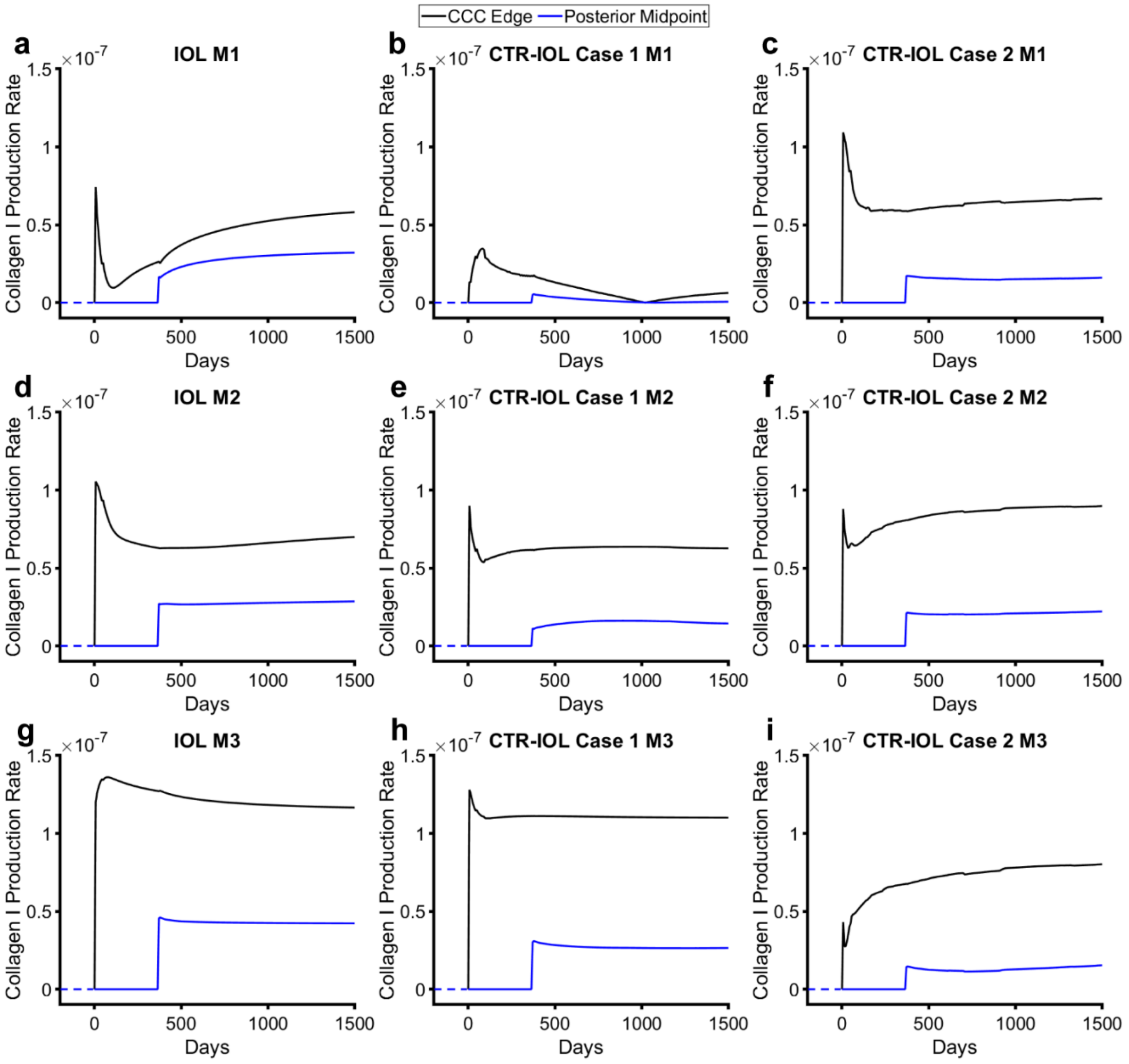

Supplementary Figure S1 Post-surgical lens capsule mass production rates of type I collagen for the FE-G&R models over time after cataract surgery. The three models are: **a**,**d**,**g** the post-surgical lens capsule with implanted IOL, **b**,**e**,**h** the post-surgical lens capsule with implanted CTR and IOL where the IOL haptics are aligned horizontally (Case 1), and **c**,**f**,**i** the post-surgical lens capsule with implanted CTR and IOL where the IOL haptics are aligned vertically (Case 2). Mass production rates are shown at the CCC edge and posterior midpoint along M1 (top row), M2 (middle row), and M3 (bottom row). Homeostatic values are shown for comparison (dashed black line).

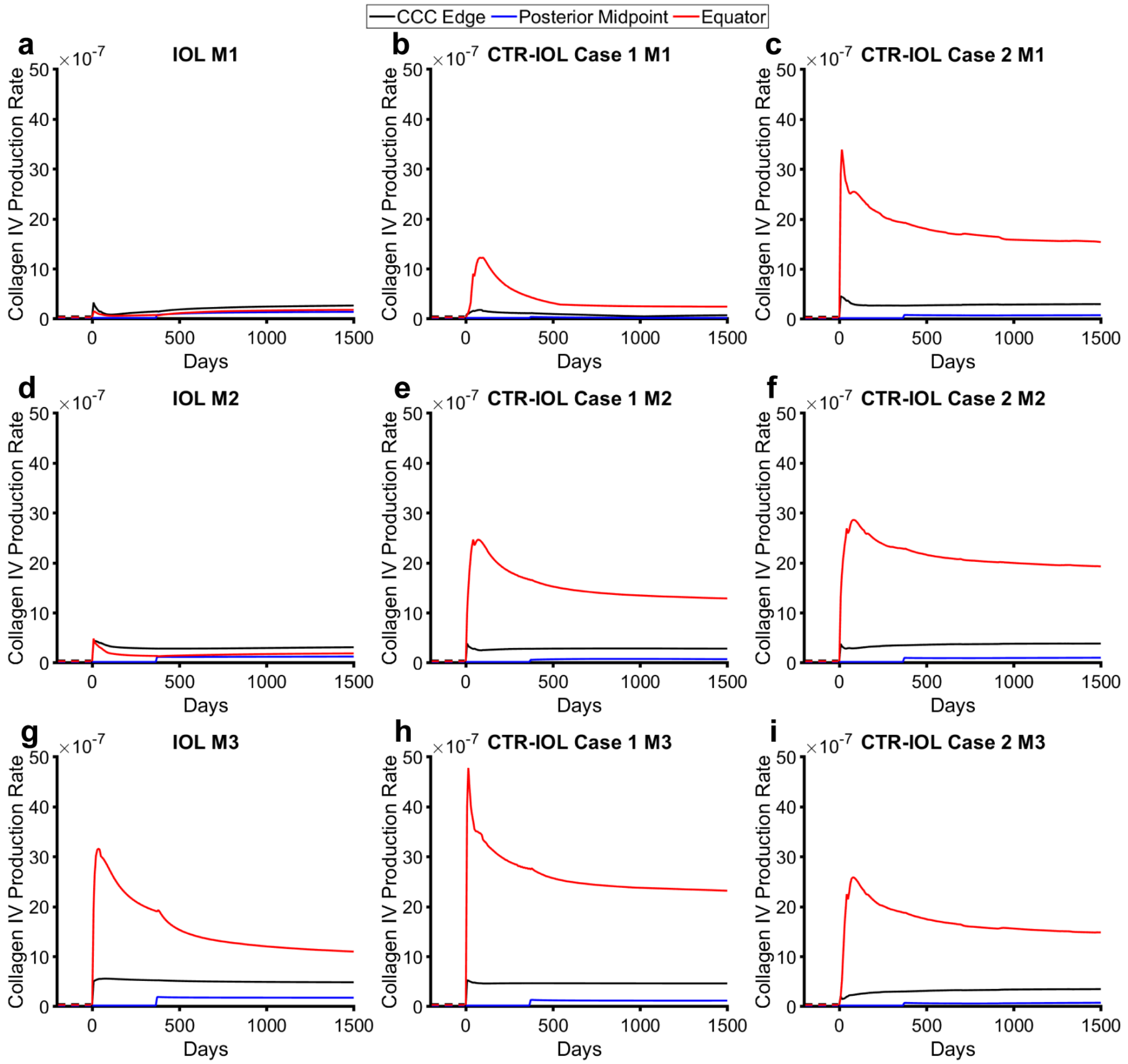

Supplementary Figure S2 Post-surgical lens capsule mass production rates of type IV collagen for the FE-G&R models over time after cataract surgery. The three models are: (**a,d,g**) the post-surgical lens capsule with implanted IOL, (**b,e,h**) the post-surgical lens capsule with implanted CTR and IOL where the IOL haptics are aligned horizontally, and (**c,f,i**) the post-surgical lens capsule with implanted CTR and IOL where the IOL haptics are aligned vertically. Mass production rates are shown at the CCC edge, posterior midpoint, and equator along M1 (top row), M2 (middle row), and M3 (bottom row). Homeostatic values (dashed line at time less than zero) are shown for comparison.

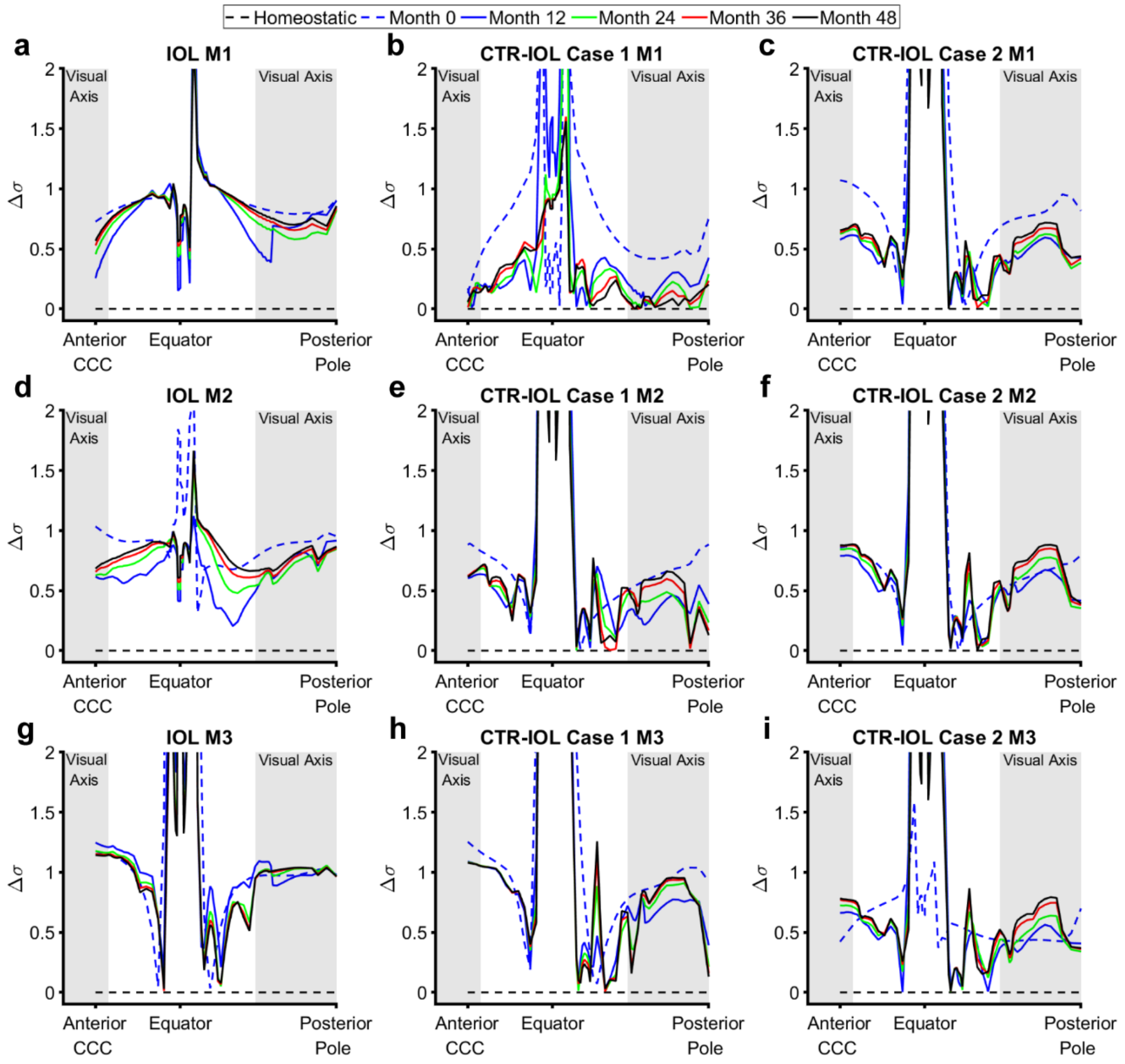

Supplementary Figure S3 The change in post-surgical lens capsule stress from homeostatic ($\Delta\sigma$) of the FE-G&R models at select simulation times up to the maximum of four years after cataract surgery. The three models are: a,d,g the post-surgical lens capsule with implanted IOL, b,e,h the post-surgical lens capsule with implanted CTR and IOL where the IOL haptics are aligned horizontally (Case 1), and c,f,i the post-surgical lens capsule with implanted CTR and IOL where the IOL haptics are aligned vertically (Case 2). Change in stress at each time is shown from the CCC edge to the posterior pole along M1 (top row), M2 (middle row), and M3 (bottom row). The regions of the anterior and posterior visual axes are highlighted in grey. Homeostatic values of $\Delta\sigma$ are zero by definition and are shown for comparison (dashed black line).

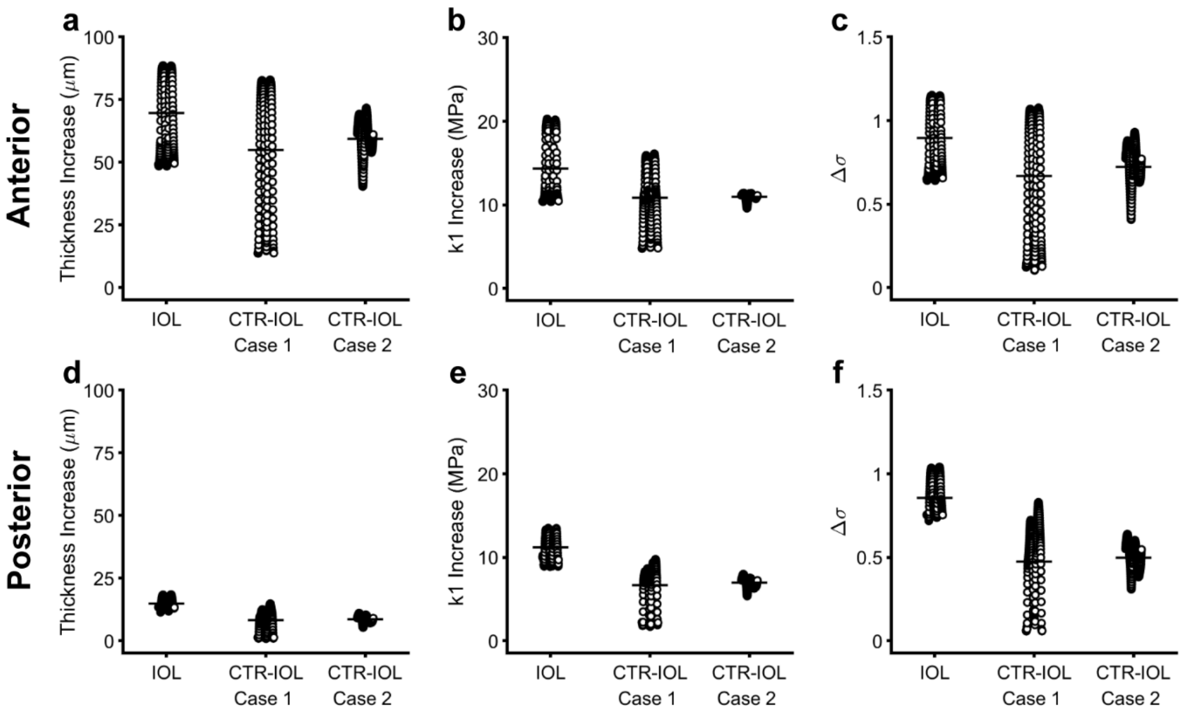

Supplementary Figure S4 Increase in thickness, stiffness, and change in stress relative to homeostatic averaged over the elements of each meridian within the visual axis of both the anterior (top row) and posterior (bottom row) portions of the post-surgical capsule models at four years after cataract surgery. a,b Increase in thickness in the a anterior and b posterior portions of the capsule along the visual axis. c,d Increase in stiffness (k1 parameter of the Holzapfel model). e,f Change in stress. Horizontal line for each model represents the mean value.

SUPPLEMENTARY VIDEOS

Supplementary Video S1 FE simulation of the IOL being placed in the post-surgical lens capsule (IOL model) at 1 week after surgery.

Supplementary Video S2 FE simulation of the CTR and IOL being placed in the post-surgical lens capsule with the IOL haptics aligned horizontally (CTR-IOL Case 1 model) at 1 week after surgery.

Supplementary Video S3 FE simulation of the CTR and IOL being placed in the post-surgical lens capsule with the IOL haptics aligned vertically (CTR-IOL Case 2 model) at 1 week after surgery.

Note: Videos are uploaded as separate ZIP files in Supplementary Material.
